# Quantitative muscle MRI in sporadic inclusion body myositis (sIBM): A prospective cohort study

**DOI:** 10.3233/JND-240053

**Published:** 2024-09-03

**Authors:** Lara Schlaffke, Robert Rehmann, Martijn Froeling, Anne-Katrin Güttsches, Matthias Vorgerd, Elena Enax-Krumova, Johannes Forsting

**Affiliations:** a Department of Neurology, BG-University Hospital Bergmannsheil, Ruhr-University Bochum, Bochum, Germany; b Department of Neurology, Klinikum Dortmund, University Witten-Herdecke, Dortmund, Germany; c Department of Radiology, University Medical Centre Utrecht, Utrecht, The Netherlands; d Heimer Institute for Muscle Research, BG-University Hospital Bergmannsheil, Bochum, Germany

**Keywords:** Diffusion tensor imaging, sporadic inclusion body myositis, quantitative muscle MRI, fat fraction, water T2 relaxation time

## Abstract

**Background:**

Sporadic inclusion body myositis (sIBM) is the predominant idiopathic inflammatory myopathy (IIM) in older people. Limitations of classical clinical assessments have been discussed as possible explanations for failed clinical trials, underlining the need for more sensitive outcome measures. Quantitative muscle MRI (qMRI) is a promising candidate for evaluating and monitoring sIBM.

**Objective:**

Longitudinal assessment of qMRI in sIBM patients.

**Methods:**

We evaluated fifteen lower extremity muscles of 12 sIBM patients (5 females, mean age 69.6, BMI 27.8) and 12 healthy age- and gender-matched controls. Seven patients and matched controls underwent a follow-up evaluation after one year. Clinical assessment included testing for muscle strength with Quick Motor Function Measure (QMFM), IBM functional rating scale (IBM-FRS), and gait analysis (6-minute walking distance). 3T-MRI scans of the lower extremities were performed, including a Dixon-based sequence, T2 mapping and Diffusion Tensor Imaging. The qMRI-values fat-fraction (FF), water T2 relaxation time (wT2), fractional anisotropy (FA), mean diffusivity (MD), axial diffusivity (*λ*_1_), and radial diffusivity (RD) were analysed.

**Results:**

Compared to healthy controls, significant differences for all qMRI parameters averaged over all muscles were found in sIBM using a MANOVA (*p* < 0.001). In low-fat muscles (FF < 10%), a significant increase of wT2 and FA with an accompanying decrease of MD, *λ*_1_, and RD was observed (*p*≤0.020). The highest correlation with clinical assessments was found for wT2 values in thigh muscles (*r*≤–0.634). Significant changes of FF (+3.0%), wT2 (+0.6 ms), MD (–0.04 10^-3^mm^2^/s), *λ*_1_ (–0.05 10^-3^mm^2^/s), and RD (–0.03 10^-3^mm^2^/s) were observed in the longitudinal evaluation of sIBM patients (*p*≤0.001). FA showed no significant change (*p* = 0.242).

**Conclusion:**

qMRI metrics correlate with clinical findings and can reflect different ongoing pathophysiological mechanisms. While wT2 is an emerging marker of disease activity, the role of diffusion metrics, possibly reflecting changes in fibre size and intracellular deposits, remains subject to further investigations.

## Abbreviations and acronyms:

6-MWD: 6-minute walking distance
10-MWT: 10-meter walk test
EPG: Extended phase graph
FA: Fractional anisotropy
FF: Fat fraction
FOV: Field of view
sIBM: Sporadic inclusion body myositis
IBM-FRS: Inclusion Body Myositis-Functional Rating Scale
IDEAL: Iterative decomposition of water and fat with echo asymmetry and least-squares estimation
iWLLS: Iterative weighted linear least squares
MD: Mean diffusivity
mDTI: Muscle diffusion tensor imaging
MRC: Medical Research Council
NMD: Neuromuscular diseases
NSS: Neuromuscular symptom score
PCA: Principal component analysis
QMFM: Quick Motor Function Measure
qMRI: Quantitative magnetic resonance imaging
RD: Radial diffusivity
SNR: Signal-to-noise ratio

## INTRODUCTION

Sporadic inclusion body myositis (sIBM), the most common idiopathic inflammatory myopathy (IIM) in people older than 50 years, exhibits a prevalence of up to 3/100.000 [1]. While the mean age at symptom onset typically falls around 65 years, diagnostic delays result in an average interval of approximately five years between symptom onset and diagnosis [2]. This condition primarily leads to progressive muscle weakness, especially in knee extension and finger flexion [3]. It may also affect oropharyngeal and oesophageal musculature, resulting in dysphagia [4]. The progression of weakness in sIBM leads to a gradual decline of hand function, increased susceptibility to falls, and, eventually, a loss of independent mobility [5]. Diagnosing sIBM typically involves a skeletal muscle biopsy, revealing distinct histopathological features such as rimmed vacuoles, endomysial inflammatory infiltrates and intracellular protein aggregates [3, 6]. The underlying pathophysiology remains controversial since muscle biopsies of affected individuals show both inflammatory and degenerative changes [7]. One theory proposes an initial inflammatory process resulting in myodegeneration, while another suggests that protein aggregation initiates secondary inflammation [8]. The common final pathway of both theories consists of progressive degeneration and fat replacement of muscle tissue. The uncertainty regarding the exact etiopathogenesis of sIBM presents a significant challenge in developing effective treatments. In the past decades, several clinical trials, including conventional and advanced immunomodulators, have failed to show clinical benefits despite promising results in the early phases in some cases [9– 14]. In this context, limitations of classic clinical assessments, such as subjectivity of ratings and wide inter-rater and intra-rater variability, have been considered [15]. Different suitable outcome measures are discussed alongside classic clinical evaluation to quantify disease progression and accurately document therapeutic efficacy [15, 16].

Quantitative MRI (qMRI) offers a non-invasive tool for assessing muscular injuries, inflammation, and degeneration in neuromuscular diseases (NMD) [17– 19]. Particularly, Dixon-based sequences enhance the ability to quantify and monitor fat replacement [20]. Elevated levels in quantitative water T2 relaxation times indicate myoedema and inflammation [21]. Muscle diffusion tensor imaging (mDTI) provides insights into the underlying pathophysiological processes by tracking the movement of water molecules [22, 23]. Recent studies were able to correlate mDTI parameters with histological findings [24, 25]. The precision and reliability of metrics derived from qMRI surpass the semiquantitative visual inspections [26]. Notably, as detected by Dixon-based sequences, an increase in fat fraction can precede muscle function decline and serve as predictive markers [27]. In some defined NMDs, such as Duchenne muscular dystrophy or calpainopathies, water T2 relaxation times and diffusion parameter changes have been described even before the onset of fat replacement [28, 29]. Given these advantages, qMRI has emerged as a promising technique for evaluating NMD.

Previous MRI studies in sIBM successfully identified a selective pattern of muscle involvement, predominantly affecting the vastus lateralis, gastrocnemius medialis, and flexor digitorum [30]. However, quantitative MRI studies showed that the sartorius and gracilis muscles in the thigh and both heads of gastrocnemius in the leg exhibited the most severe affection [31]. Furthermore, qMRI parameters, including fat fraction (FF) and water T2 relaxation time, reflected a clinical deterioration of muscle function [32, 33]. This study aims to extend these findings using an established quantitative MRI protocol in sIBM, including mDTI and longitudinal analysis after one-year follow-up [34].

## METHODS

### Study population

**Fig. 1:**
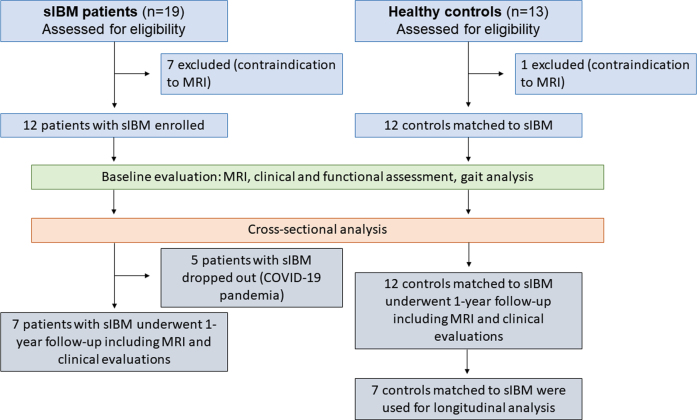
Flowchart of study procedure.

In total, 12 individuals with probable (*n* = 1) or definite (*n* = 11) sIBM according to the ENMC criteria (5 females, mean age 69.6±6.4 (57– 78) years; BMI 27.8±3.7) and 12 age-matched healthy volunteers (6 females, mean age 59.9±5.0 (53– 68) years, BMI 24.3±2.4) participated in this study [3, 35]. A follow-up examination was conducted in 7 patients and 7 matched controls within 13 months±4 weeks after enrolment. This study was approved by the local ethics committee of the Ruhr-Univerity Bochum (No: 15-5281), and written informed consent was obtained from all participants. The study protocol is summarised in Fig. 1. The exclusion criteria for healthy volunteers included a medical history of NMD and lower extremity injuries within the 12 months before study enrolment.

### Clinical assessments

Experienced clinicians assessed muscle strength using the Medical Research Council (MRC) scale and Quick Motor Function Measure (QMFM) [36]. The MRC scale is a frequently utilised tool for assessing muscle strength across a spectrum from 0 to 5. The QMFM comprehensively evaluates diverse components of motor function, including strength and coordination, at a range between 0 and 64 points [36]. Physical function was assessed using the Inclusion Body Myositis-Functional Rating Scale (IBM-FRS) [37]. The IBM-FRS assesses daily activities using ten questions with a 0 to 4 scale, resulting in a score of 0 to 40 points. In both QMFM and IBM-FRS, lower scores suggest worse performance. An experienced medical technical assistant conducted the 6-minute walking distance (6MWD) for mobility assessment in ambulant individuals.

### MRI acquisition and sequences

Participants were positioned supinely with their feet first. To ensure comfort and minimise movement, cushions supported the participants’ knees, and sandbags were strategically placed around their feet. A Philips 3.0T Achieva MR system and a 16CH Torso XL coil were used to capture scans from both legs. These scans were oriented perpendicular to the femur and tibia bone. The thigh area, stretching from hip to knee, was divided into two fields of view (FOV) of 480×276×150 mm^3^ along the z-axis. There was a 30 mm overlap between these FOVs to ensure comprehensive coverage and continuity in imaging. The proximal edge was positioned in the pelvic region. For the calf region, a single FOV measuring 480×276×150 mm^3^ was used. The proximal edge of the single FOV used for the calf region was positioned 60 mm below the tibial plateau. The scanning protocol included the following sequences [34]:

1. A 4-point Dixon-based sequence with a voxel size of 1.5x1.5x6.0mm^3^, TR/TE 210/2.6, 3.36, 4.12, 4.88 ms, flip angle 8°, SENSE: 2).
2. A multi-echo spin-echo (MESE) sequence for quantitative water mapping including 17 echoes and Cartesian k-space sampling with a voxel size of 3.0×3.0×6.0 mm^3^, 6 mm gap, TR/TE 4598/17x*Δ*7.6 ms, flip angle 90/180°, SENSE: 2).
3. A diffusion-weighted spin-echo EPI with a voxel size 3.0×3.0×6.0 mm^3^, TR/TE 5000/57 ms, SPAIR/SPIR fat suppression, SENSE: 1.9, 42 gradient directions with eight different b-values (0–600).

An additional noise scan was acquired using the same imaging parameters as the DWI, except without RF power and gradients (only acquisition channels open). The total duration of the scanning process was approximately 36 minutes.

### Data pre-processing

Data were pre-processed using QMRITools (www.qmritools.com), adhering to previously established protocols [34, 38]. Initially, the diffusion data underwent denoising using a principal component analysis (PCA) [39]. Data for each leg were separately registered to correct for subject motion and eddy currents. Subsequently, tensor calculations were performed, incorporating intravoxel incoherent motion (IVIM) and utilising an iterative weighted linear least squares (iWLLS) algorithm [40, 41]. The *iterative* decomposition of water and fat with echo asymmetry and least-squares estimation (IDEAL) method was applied to the Dixon-based data, assuming a single T2* decay and resulting in separate water and fat maps [42]. These water maps were then used for manual segmentation. The T2-mapping data were analysed using an extended phase graph (EPG) fitting approach [43].

### Muscle segmentation

Muscle segmentation included eight thigh muscles (vastus lateralis, vastus medialis, rectus femoris, semimembranosus, semitendinosus, biceps femoris (long and short head), sartorius, and gracilis) and seven leg muscles (gastrocnemius medialis and lateralis, soleus, tibialis anterior, peroneus, extensor digitorum and tibialis posterior) using a semiautomatic segmentation approach [44]. Subsequently, the segmentations in both legs were refined by an experienced rater using 3D-slicer 4.4.0 (https://www.slicer.org). Muscles with a volume of less than 5 cm^3^ were excluded from further analysis.

For the longitudinal data, to ensure consistency across repeated scans, a combined rigid, affine and b-spline registration in QMRITools was used to align corresponding areas, resulting in adjusted segmentation masks [17, 38].

Segmentations were registered to T2 and DTI data to adjust for small motions between sequences and image distortions using sequential rigid and b-spline transformations with the elastix software (https://elastix.lumc.nl) [45]. Water T2 relaxation time and proton density fat fraction (FF) averages were calculated across all slices. The signal-to-noise ratio (SNR) was calculated by dividing the local average signal by the local noise sigma [46]. For analysis of diffusion data, the segmentation masks were smoothed and eroded by one voxel to avoid partial volume effects of non-muscular tissue and registered to the diffusion space to extract the diffusion metrics of fractional anisotropy (FA), mean diffusivity (MD), axial diffusivity (*λ*_1_), and radial diffusivity (RD) for each muscle.

### Statistical analysis

To examine the differences in qMRI metrics between the patient and control group at baseline, a general linear model (GLM) was employed, considering the patient/control group, body side, and muscle group as fixed factors. A repeated-measures MANOVA was utilised to assess the changes in the qMRI metrics between baseline and follow-up in the patient and control group. Muscles were functionally clustered in the following muscle groups: hamstrings (biceps femoris, semitendinosus, semimembranosus), quadriceps (rectus femoris, vastus medialis, vastus lateralis), adductors (gracilis, sartorius) in the thigh, anterior group (extensor digitorum, peroneal group, tibialis anterior), and posterior group (gastrocnemius medialis, gastrocnemius lateralis, soleus, tibialis posterior) in the leg. Post-hoc t-tests were performed for each muscle group using Sidak correction to adjust for multiple comparisons. For diffusion metrics analysis, muscles with an FF > 80% and an SNR below ten, indicating poor data quality, were excluded [28, 47, 48]. In a subsequent cross-sectional study, all muscles with an FF > 10% were excluded to evaluate water T2 times and diffusion metrics between low-fat muscles in patients and controls at baseline [49].

The Wilcoxon signed-rank test, quantified by z-scores, was used to assess changes between clinical data at baseline and follow-up.

MRI outcome parameters were compared to clinical outcome measures by calculating compound scores for all thigh and leg muscles, considering each muscle’s segmentation mask volume. Spearman correlation coefficients were used to explore relationships between MRI parameters, patient clinical outcomes, and changes in these parameters. Furthermore, water T2 time and main diffusion metrics were correlated with FF change over time. The interpretation of the observed correlation coefficient followed the guidelines proposed by Schober et al. without adjustment of p-values for multiple testing. The correlation strength categories were defined as follows: 0.00– 0.10, indicating a negligible correlation; 0.10– 0.39, suggesting a weak correlation; 0.40– 0.69, indicating a moderate correlation; 0.70– 0.89, suggesting a strong correlation; and 0.90– 1.00, indicating a robust correlation [50].

All statistical analyses were conducted using IBM SPSS V28, with a significance level of *p* < 0.05.

## RESULTS

### Clinical characteristics of patients

Table 1 provides an overview of clinical data for the patients. The mean disease duration at baseline in the study cohort was 8.9±3.6 years, ranging from 2 – 14 years. QMFM results ranged from 4 to 49 (mean 35.4±14.2), while IBM-FRS ranged from 14 to 36 (mean 27.3±7.6). Due to data collection during the COVID-19 pandemic, only seven sIBM patients underwent a follow-up assessment (see Fig. 1). Within the clinical outcome measures, significant changes between baseline and follow-up in the patient group were found for the QMFM (*p* = 0.046) but not for IBM-FRS (*p* = 0.443) and 6MWD (*p* = 0.715).

**Table 1:** Demographic and clinical data of patients with sporadic inclusion body myositis (sIBM)

Patient	Sex	BMI (kg/m^2^)	Age (years)	Disease duration (years)	ENMC criteria	IBM-FRS	QMFM	6-MWD (meter)	Follow-up	Medication
1	Male	23.7	57	12	Definite	16	4	–	Yes	IVIG
2	Male	27.8	62	7	Probable	30	48	428	Yes	IVIG*
3	Male	24.2	66	12	Definite	17	29	165	Yes	IVIG
4	Male	26.6	73	6	Definite	28	35	287	Yes	IVIG
5	Male	24.2	69	9	Definite	34	49	425	No	MTX
6	Male	30.2	73	8	Definite	31	35	500	No	IVIG
7	Male	24.6	78	8	Definite	36	46	590	No	None
8	Female	30.0	58	13	Definite	29	45	420	Yes	IVIG
9	Female	26.1	70	14	Definite	14	17	–	Yes	None
10	Female	36.5	70	11	Definite	25	26	178	Yes	IVIG
11	Female	24.6	71	4	Definite	33	48	460	No	None
12	Female	22.7	76	2	Definite	34	43	320	No	Kortison

^*^Treatment suspension in follow-up following patient request. ENMC – European Neuromuscular Center; IBM-FRS – Inclusion Body Myositis Functional Rating Scale; QMFM – Quick Motor Function Measure; 6-MWD – 6-Minute Walking Distance; MTX – Methotrexate; IVIG – Intravenous Immunglobuline.

### MRI findings

**Fig. 2:**
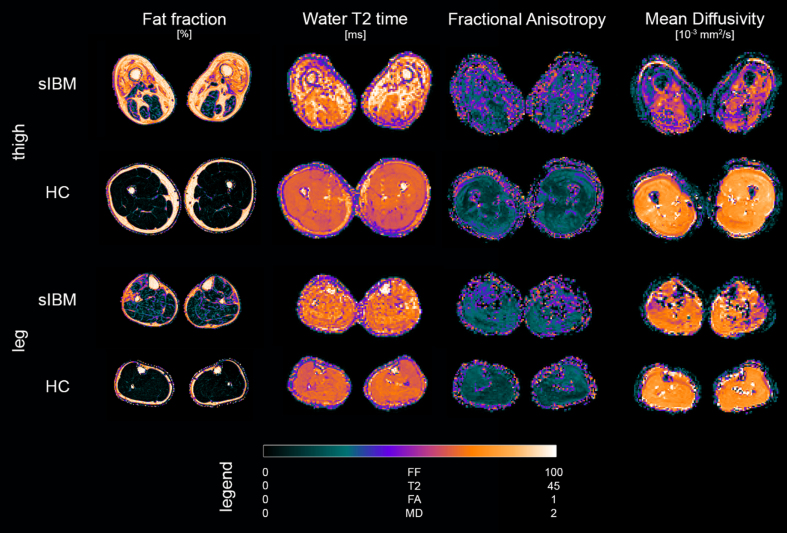
Overview of parameter maps for fat fraction (FF), water T2 relaxation time (T2), fractional anisotropy (FA), and mean diffusivity (MD) of representative sporadic inclusion body myositis patient (sIBM) and healthy control (HC).

Scans were successfully conducted on all participants. Figure 2 gives an exemplary overview of parameter maps of qMRI metrics in a representative patient and healthy control.

#### Differences in qMRI metrics between patient and control group at baseline

**Fig. 3:**
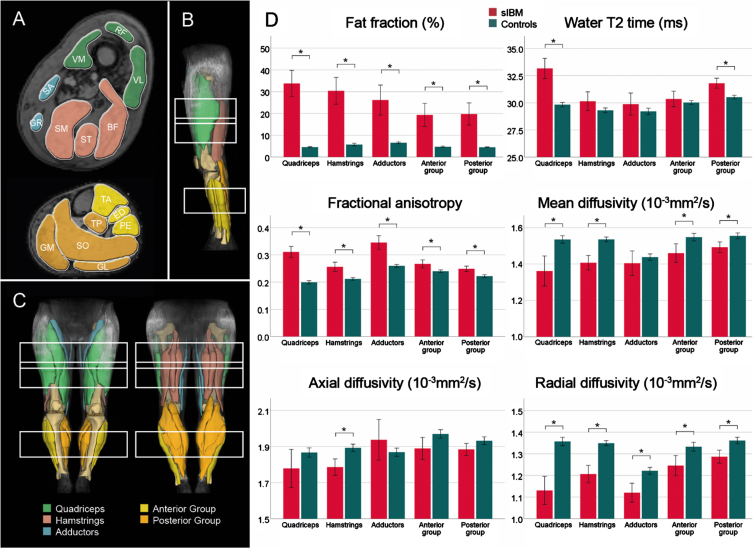
Overview of segmented muscles and corresponding muscle groups in a representative participant (A – cross-sectional; B – side view, C – front and back view). Bar plots show mean qMRI metrics for patients with sporadic inclusion body myositis (sIBM) and control group (D). The lines show the 95% -interval of confidence. *adjusted p < 0.05. *BF* = *biceps femoris; ED* = *extensor digitorum longus; GM* = *gastrocnemius medialis; GL* = *gastrocnemius lateralis; GR* = *gracilis; PE* = *peroneal group; RF* = *rectus femoris; SA* = *sartorius; SO* = *soleus*; *SM* = *semimembranosus; ST* = *semitendinosus; TA* = *tibialis anterior; TP* = *tibialis posterior; VL* = *vastus lateralis; VM* = *vastus medialis*.

The bar graphs in Fig. 3 illustrate the differences between the patient and control group at baseline. Significant main effects of FF and water T2 relaxation time were found between the patient and control group (*p* < 0.001). FF was significantly higher in all muscle groups of patients (*p* < 0.001), while water T2 time of patients was only higher in the quadriceps (*p* < 0.001) in the thigh and the posterior group of the leg (*p* < 0.001). In the analysis of diffusion metrics, all parameters showed significant differences between study groups (*p*≤0.001). While a significantly higher FA and lower RD were observed in all muscle groups (*p*≤0.005) for all patients, MD was significantly lower in all muscle groups (*p*≤0.001) except for the adductors (*p* = 0.337). *λ*_1_ was only significantly lower in hamstring muscles (*p*≤0.001) in the thigh in sIBM patients compared to controls.

**Fig. 4:**
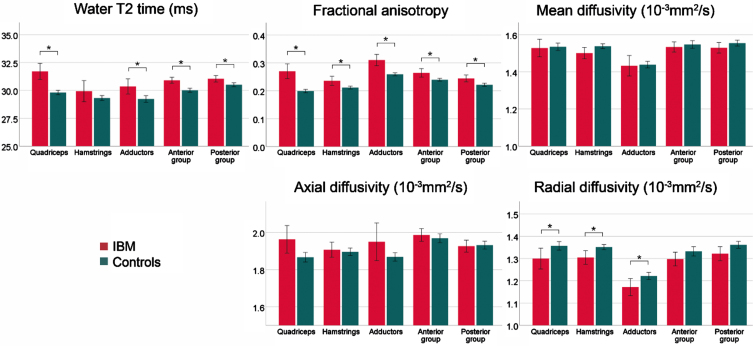
Overview of mean qMRI metrics low-fat muscles (FF < 10%) for the different muscle groups of patients with sporadic inclusion body myositis (sIBM) and control group. The lines show the 95% -interval of confidence. *adjusted p < 0.05.

After the exclusion of muscles with a FF > 10%, significant differences for water T2 (*p*≤0.001), FA (*p* < 0.001), MD (*p* = 0.020), *λ*_1_ (*p* = 0.004), and RD (*p* < 0.001) were found between study groups. Post-hoc analysis for each muscle group confirmed a significantly higher water T2 in all muscle groups except for hamstrings (*p* = 0.223). FA was higher in all muscle groups of sIBM patients compared to controls, while a lower RD was observed in all thigh muscles (*p*≤0.010). Post-hoc tests for MD and *λ*_1_ showed no differences between groups. The bar graphs in Fig. 4 show the differences between both study groups in low-fat muscles.

#### Correlations of qMRI metrics with clinical assessments at baseline

All correlations between qMRI metrics and clinical examinations at baseline are displayed in Table 2. qMRI metrics and age did not correlate significantly. MD in the thigh correlated significantly negatively with the disease duration (*r* = –0.635, *p* = 0.026). A moderate correlation between FF of thigh muscles and disease duration was observed but did not reach statistical significance (*r* = 0.547, *p* = 0.065). Further, there were significant negative correlations of FF and water T2 and a positive correlation of MD in the thigh with IBM-FRS and QMFM. The 6-MWD correlated significantly only with water T2 in the thigh (*r* = –0.721, *p* = 0.019).

**Table 2:** Spearman correlation coefficients of compound score of qMRI parameters fat fraction (FF), fractional anisotropy (FA), mean diffusivity (MD), and T2 relaxation time (T2) and clinical outcome measures in patients with sporadic inclusion body myositis (n = 12) at baseline (t_0_). ^*^p < 0.05, ^**^p < 0.01

	Thigh muscles				Calf muscles			
	FF (t_0_)	T2 (t_0_)	FA (t_0_)	MD (t_0_)	FF (t_0_)	T2 (t_0_)	FA (t_0_)	MD (t_0_)
Age (y)	–0.302	–0.123	0.098	0.372	–0.232	0.070	0.302	0.319
DD (y)	0.547	0.196	0.014	–0.635*	0.323	0.400	–0.330	–0.302
IBM-FRS	–0.655*	–0.634*	–0.441	0.711**	–0.385	–0.235	0.007	0.214
QMFM	–0.635*	–0.796*	–0.670*	0.695*	–0.428	–0.596*	–0.095	0.242
6-MWD [m]	–0.176	–0.721*	–0.382	0.164	–0.176	–0.236	–0.321	0.406

DD – Disease Duration; IBM-FRS – Inclusion Body Myositis Functional Rating Scale; QMFM – Quick Motor Function Measure; 6-MWD – 6-Minute Walking Distance.

#### Changes in qMRI metrics between baseline and follow-up

**Fig. 5:**
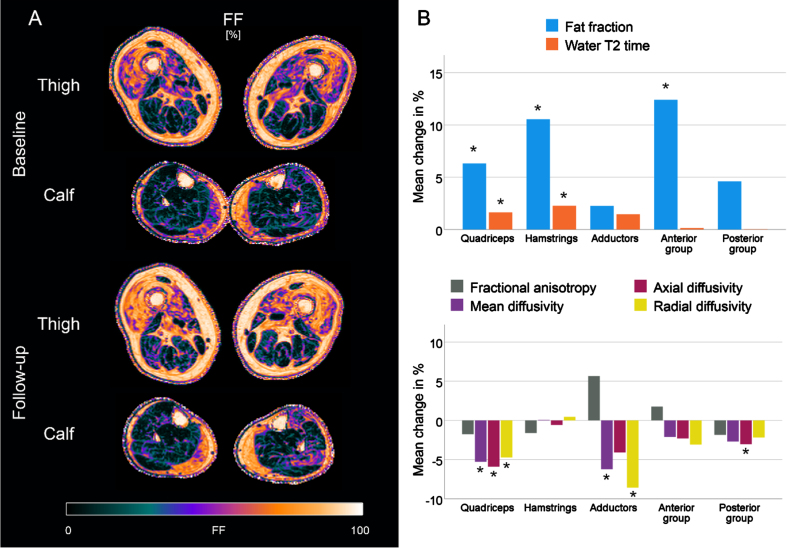
Fat fraction (FF) maps of a representative patient with sporadic inclusion body myositis (sIBM) at baseline and follow-up (A). Bar plots showing the relative mean changes of qMRI metrics in IBM patients between baseline and follow-up, normalized to the mean of both measurements. *adjusted p < 0.05.

Table 3 summarises the qMRI data over time. The relative changes in qMRI metrics, calculated as a percentage of the mean value of both measurements, are illustrated in the bar plots in Fig. 5B. sIBM patients showed an increase of 3.0% in mean fat fraction (95% -CI: 2.2 to 3.9%; *p* < 0.001), with the highest changes in the quadriceps (3.9%) and the hamstrings (4.2%). Water T2 times also increased significantly during the study course (+0.6 ms; 95% -CI: 0.3 to 0.9 ms; *p* < 0.001). At the level of muscle groups, significant changes were observed in the quadriceps (+1.3 ms; *p* < 0.001) and the hamstrings (+1.2 ms; *p* < 0.001). For the diffusion, a significant decrease was observed for MD, *λ*_1_, and RD over all muscles (*p* < 0.001). Changes in these parameters seem mainly driven by a significant reduction in the quadriceps (*p* < 0.001). In contrast, no changes in the hamstrings and anterior group of leg muscles were observed. No significant changes in FA were found. Changes in the control group were small (FF:+0.1%; water T2:+0.1 ms; FA:+0.00; MD:+0.01 10^-3^mm^2^/s), although some significant main effects were observed (see Table S3).

**Table 3:** qMRI metrics over time for patients with sporadic inclusion body myositis

qMRI metrics	*N*	Baseline (SE)	Change [95% -CI]	*p*-value
**Fat fraction (%)**	195	32.4 (1.9)	+3.0 [2.2;3.9]	<0.001*
Quadriceps		44.1 (4.2)	+3.9 [2.1;5.7]	<0.001*
Hamstrings		37.9 (4.0)	+4.2 [2.5;6.0]	<0.001*
Adductors		32.3 (6.0)	+2.5 [-0.1;5.1]	0.064
Anterior Group		27.8 (4.1)	+3.6 [1.8;5.4]	<0.001*
Posterior Group		23.3 (3.5)	+1.2 [-0.3;2.8]	0.115
**Water T2 (ms)**	195	31.3 (0.3)	+0.6 [0.3; 0.9]	<0.001*
Quadriceps		34.1 (0.6)	+1.3 [0.6;1.9]	<0.001*
Hamstrings		29.9 (0.6)	+1.2 [0.6;1.9]	<0.001*
Adductors		29.3 (0.9)	+0.7 [-0.3;1.6]	0.168
Anterior Group		30.0 (0.6)	+0.2 [-0.5;0.8]	0.618
Posterior Group		32.0 (0.5)	+0.0 [-0.5;0.6]	0.900
**FA**	176	0.29 (0.01)	+0.00 [-0.01;0.01]	0.242
Quadriceps		0.33 (0.01)	+0.00 [-0.02;0.01]	0.619
Hamstrings		0.28 (0.01)	-0.01 [-0.02;0.01]	0.459
Adductors		0.35 (0.02)	+0.02 [0.00;0.05]	0.072
Anterior Group		0.29 (0.01)	+0.01 [-0.01;0.03]	0.171
Posterior Group		0.25 (0.01)	+0.00 [-0.01;0.01]	0.970
**MD (10^**-3**^ mm^**2**^/s)**	176	1.39 (0.02)	-0.04 [-0.06;-0.02]	<0.001*
Quadriceps		1.27 (0.04)	-0.07 [-0.11;-0.02]	0.009*
Hamstrings		1.34 (0.04)	-0.01 [-0.06;0.04]	0.768
Adductors		1.44 (0.06)	-0.09 [-0.16;-0.01]	0.021*
Anterior Group		1.41 (0.04)	-0.02 [-0.07;0.02]	0.330
Posterior Group		1.45 (0.03)	-0.04 [-0.08;0.00]	0.073
**AD (10^**-3**^ mm^**2**^/s)**	176	1.81 (0.02)	-0.05 [-0.08;-0.03]	<0.001*
Quadriceps		1.69 (0.05)	-0.10 [-0.16;-0.03]	0.003*
Hamstrings		1.73 (0.05)	-0.01 [-0.08;0.05]	0.674
Adductors		2.01 (0.08)	-0.08 [-0.18;0.02]	0.105
Anterior Group		1.85 (0.05)	-0.04 [-0.10;0.02]	0.179
Posterior Group		1.88 (0.04)	-0.06 [-0.11;0.00]	0.036*
**RD (10^**-3**^ mm^**2**^/s)**	176		-0.03 [-0.05; -0.02]	<0.001*
Quadriceps		1.04 (0.03)	-0.05 [-0.09;-0.01]	0.013*
Hamstrings		1.14 (0.04)	+0.00 [-0.04;0.04]	0.878
Adductors		1.13 (0.05)	-0.09 [-0.15;-0.04]	0.001*
Anterior Group		1.19 (0.03)	-0.03 [-0.06;0.01]	0.125
Posterior Group		1.28 (0.03)	-0.03 [-0.06;0.01]	0.097

#### Correlations of the longitudinal evaluation of qMRI metrics and clinical examinations

Correlations of changes in qMRI metrics and changes in clinical assessments are summarised in Table S2. A significant strong negative correlation between changes of water T2 in the thigh muscles and alterations in IBM-FRS was found (*r*=-0.793, *p* = 0.033). Strong negative correlations were also observed for changes of 6-MWD and water T2 in the thigh (*r*=-0.820, *p* = 0.089) and leg muscles (*r*=-0.830, *p* = 0.082). Similar effects were observed for the change of QMFM and the change of FF in the thigh (*r*=-0.679, *p* = 0.094) and leg (*r*=-0.714, *p* = 0.071), but statistical significance was not reached, probably due to the small cohort size. No significant correlations were found between the FF change over time and water T2 (*r* = 0.067, *p* = 0.354) and MD (*r* = 0.013, *p* = 0.858). Nevertheless, a significant correlation emerged between FA at baseline and the FF change over time (*r* = 0.287, *p* < 0.001).

## DISCUSSION

In this study, we extended the findings of previous studies underlying the feasibility and utility of qMRI in the assessment of sIBM. qMRI metrics capture the disease-specific patterns of muscle involvement in terms of fat replacement and correlate with clinical outcome measures and their change over time [31–33]. These findings underscore the potential of qMRI metrics as a biomarker for assessing and monitoring muscle tissue functionality in sIBM.

The quantification of muscular FF using Dixon-based sequences has been previously shown to identify the known disease-specific pattern, predominantly affecting the quadriceps in the thigh and the gastrocnemius medialis in the leg [30, 31]. Furthermore, FF has been shown to detect subtle changes in muscle fat content in sIBM patients during follow-up studies [32, 33]. The FF changes in this study, with an average increase of 3.0%, align with these previous observations, showing an FF increase between 2.4% and 3.3% over one year. The potential of qMRI to detect subtle variations in muscle fat content shows considerable promise for detecting disease-specific patterns of fat replacement and accurate monitoring of disease progression and treatment effectiveness. Compared to the other qMRI metrics in our study, changes in FF exhibit proportionally higher values, underlining its value in monitoring disease progression. However, fat replacement is the final stage of different pathophysiological mechanisms in NMD and is non-reversible. Water T2 relaxation time and diffusion metrics could detect early pathophysiological changes in NMD before the onset of irreversible fat replacement, thereby detecting muscle tissue still to be saved [29, 49].

Water T2 relaxation time has been recently shown to correlate with various histopathological findings in skeletal muscle biopsies, especially in inflammatory myopathies [24, 51]. These correlations include inflammatory cell infiltration, degree of fibre size variation and amount of connective tissue [51]. Therefore, water T2 relaxation time has been identified as a potential marker of disease activity in different NMD, especially in sIBM [32, 33, 52]. This study found higher water T2 values in sIBM patients compared to healthy controls, suggesting ongoing inflammation, especially in the quadriceps and the posterior compartment of the leg. Notably, higher water T2 values were found in low-fat muscles across all muscle groups except for the hamstrings, indicating the potential utility of water T2 relaxation time in the early detection of disease-specific alterations. Previous research has described early changes in water T2 values preceding marked intramuscular fat replacement, supporting the hypothesis of a primary inflammatory process followed by a secondary degeneration [8, 33]. In our study, the potential of water T2 relaxation time as a marker for disease activity is emphasised by the strong correlations of water T2 values in the predominantly affected thigh muscles and clinical outcome parameters at baseline, as well as the correlation between water T2 changes in the thigh with changes in clinical assessments during follow-up. Interestingly, we found a significant increase in water T2 relaxation time in the quadriceps and hamstrings, contrasting a previous study in a large cohort of sIBM patients [32]. Despite the same mean disease durations in both studies (8.9 years), Laurent et al. found a significant decrease over one year [32]. In the advanced disease stages, an artificial reduction of water T2 due to ongoing fat replacement and fibrotic remodelling could occur, possibly explaining the findings of Laurent et al. [53]. At the same time, during our study, inflammatory activity seemed to increase in the thigh muscles. Other studies have also reported an increase in water T2 values in sIBM patients with comparable disease durations (7.7 years) [33]. Further investigation is needed to understand the underlying pathophysiological mechanisms of different water T2 dynamics in sIBM.

Like the water T2 relaxation time assessment, diffusion metrics have proven effective in detecting structural abnormalities in muscle tissue [25, 54]. However, the influence of fat replacement on diffusion metrics required the development of highly effective fat suppression methods, which can result in images with noise dominance [55]. This effect is particularly pronounced in tissue with high fat content, leading to anisotropic diffusion in fat-replaced muscle due to the random nature of this noise [56]. To minimise these effects, diffusion metrics with an SNR lower than ten were excluded from the statistical analysis in this study. Additionally, diffusion metrics were assessed in a secondary analysis, evaluating only low-fat muscles (FF < 10%). Compared to healthy controls, significantly elevated FA values and lower MD, *λ*_1_, and RD values were observed. An increase in FA and a decrease in MD were previously interpreted as a sign of fibre atrophy, supported by simulation experiments [48, 57]. Interestingly, the higher FA with decreasing muscle fibre size can precede MD changes, potentially explaining why post-hoc analysis of low-fat muscles showed the same effects at the muscle group level for FA and RD but not MD [57]. The observations in low-fat muscles underline that diffusion metrics display ongoing pathophysiological mechanics beyond fat replacement. However, the relatively short diffusion time used in this study may primarily illustrate intracellular processes, such as hindered diffusion due to intracellular deposits rather than fibre size [57]. Recent work in a mouse model of late-onset Pompe disease showed that muscle fibre diameter and autophagic markers correlated negatively with MD and RD [58]. The histopathological features of sIBM include endomysial inflammation, rimmed vacuoles, and intracellular protein aggregation [35]. In this context, the differences in diffusion metrics could reflect these intracellular processes, leading to a decrease in mean, axial, and radial diffusivity. The longitudinal decrease of these parameters, especially in the predominantly affected quadriceps muscles, could underscore that the differences in diffusion metrics are not only explained by fibre atrophy. In fibre hypotrophy, simulation experiments suggest an increase of FA and a decrease of MD and RD without relevant changes of *λ*_1_ [59]. The decrease of all diffusion values except for FA over time may thus indicate hindered diffusion by intracellular deposits such as vacuoles or aggregates. On the contrary, we found a weak but significant positive correlation between FA at baseline and fat fraction over time. While FA seems less sensitive in the longitudinal assessment of sIBM patients, further investigation in larger cohorts is needed to comprehensively elucidate the underlying pathophysiological mechanisms driving the observed diffusion changes.

There are some limitations to consider. Due to difficulties in recruiting healthy volunteers, the control group was, on average, nearly ten years younger than the patient group. Albeit qMRI changes in ageing are small, this could have influenced our results [60, 61]. The significant changes observed in healthy controls over time can be attributed to natural variation and measurement inaccuracies and are outside the realm of clinically relevant outcomes [62]. The overall cohort size was small compared to previous studies and not sufficiently powered to analyse differences between different treatment regimes [31]. However, only one patient changed treatment (stop of IVIG in Pat. 2). Furthermore, many drop-outs were primarily attributed to the data acquisition during the COVID-19 pandemic. Health concerns, particularly among the elderly and high-risk patients, were the main reasons to refuse follow-up. Due to the diversity of the patient cohort, limited follow-up data, and small cohort size, a final assessment of qMRI compared to clinical evaluations is still pending. Additionally, MRI acquisition and processing remain time- and resource-intensive. Implementing standardised and simplified imaging protocols and data analysis is necessary to facilitate the widespread adoption of quantitative MRI techniques and ensure comparability between different centres.

## CONCLUSION

This study showed that qMRI can reflect different pathophysiological mechanisms in sIBM patients. While FF is a marker of disease progression by illustrating fat replacement, water T2 relaxation time is a marker of disease activity, correlating with important clinical outcome measures. Water T2 times and diffusion metrics can detect early disease-specific changes in sIBM before the irreversible process of fat replacement. While diffusion metrics offer valuable insights into pathophysiology, including changes in fibre size or intracellular deposits, further research is needed to understand the underlying mechanisms fully.

## Supplementary Material

Supplementary Material

## Data Availability

The data supporting the findings of this study are available on request from the corresponding author. The data are not publicly available due to privacy or ethical restrictions.
