# Supplementary material for: Quantitative muscle MRI in sporadic inclusion body myositis (sIBM): A prospective cohort study
Source: J Neuromuscul Dis. 2024 Sep 3;11(5):997–1009. doi: 10.3233/JND-240053 (PMC11380292; doi:10.3233/JND-240053)
Supplement: Supplementary Material [file jnd-11-jnd240053-s001.docx]

**Supplements**

**Supplementary Table 1**: Scan parameters for the acquired Dixon sequences, quantitative T2, and diffusion weighted imaging (DWI).

|  | qT2 | DWI | Dixon |
| --- | --- | --- | --- |
| Sequence | MESE | SE-EPI | MS_FFE |
| FOV (LR x AP x FH mm^3^) | 480 x 276 x 150 | | |
| Acquisition matrix | 160 x 92 | 160 x 92 | 320 x 184 |
| Voxel size (mm^3^) | 3 x 3 x 6 | 3 x 3 x 6 | 1.5 x 1.5 x 6 |
| Slice gap (mm) | 6 | - | - |
| Slices | 13 | 25 | 25 |
| TR (ms) | 4598 (7650) | 5000 | 210 |
| TE (ms) | 17 x Δ 7.6 | 57 | 2.6/3.36/4.12/4.88 |
| Flip angle (°) | 90/180 |  | 8 |
| Sense factor | 2 | 1.9 | 2 |
| b‐values (number of images) | 0 | 0 (1), 1 (6), 10 (3), 25 (3), 100 (3), 200 (6), 400 (8) and 600 (12) | 0 |
| Fat suppression | - | SPAIR/SPIR | - |
| Total duration (min) | 3.08 | 4.33 | 1.33 |

**Supplementary Table 2:** Clinical tests of sporadic inclusion body myositis (sIBM) patients between time points.

*P-values are calculated using the Wilcoxon Signed Ranks Test.*

|  | **n (cases)** | **t_0_** | | | | **t_1_** | | | | **p-value** |
| --- | --- | --- | --- | --- | --- | --- | --- | --- | --- | --- |
|  |  | Mean | ± | Std | Median (IQR) | Mean | ± | Std | Median (IQR) |  |
| **IBM-FRS** | 7/7 | 22.7 | ± | 6.3 | 25 (16 - 29) | 21.6 | ± | 5.8 | 22 (17 - 27) | 0.443 |
| **QMFM** | 7/7 | 29.1 | ± | 15.4 | 29 (17 - 45) | 24.6 | ± | 15.5 | 22 (12 - 42) | 0.046 |
| **6-MWD** [m] | 5/7 | 295.6 | ± | 126.5 | 287 (172 - 424) | 299.5 | ± | 150.6 | 240 (174 - 455) | 0.715 |

*IBM-FRS – Inclusion Body Myositis Functional Rating Scale; QMFM – Quick Motor Function Measure; 6-MWD – 6-Minute Walking Distance.*

**SupplementaryTable 3:** Spearman correlation coefficients between the changes measured

after follow-up in compound score of qMRI parameters fat fraction (FF), fractional anisotropy (FA), mean diffusivity (MD), and T2 relaxation time (T2) and clinical outcome measures in patients with sporadic inclusion body myositis (n = 7). *p < 0.05

|  | **n (cases)** | **Thigh muscles** | | | | **Calf muscles** | | | |
| --- | --- | --- | --- | --- | --- | --- | --- | --- | --- |
|  |  | ∆FF | ∆T2 | ∆FA | ∆MD | ∆FF | ∆T2 | ∆FA | ∆MD |
| **∆IBM-FRS** | 7/7 | -0.234 | -0.793* | -0.180 | -0.559 | 0.505 | -0.450 | 0.144 | -0.072 |
| **∆QMFM** | 7/7 | -0.679 | -0.107 | -0.786 | 0.143 | -0.714 | -0.464 | -0.500 | 0.607 |
| **∆6-MWD [m]** | 5/7 | -0.600 | -0.900* | -0.600 | 0.000 | -0.100 | -0.800 | 0.300 | 0.500 |

*IBM-FRS – Inclusion Body Myositis Functional Rating Scale; QMFM – Quick Motor Function Measure; 6-MWD – 6-Minute Walking Distance.*

**Supplementary Table 4:** qMRI metrics over time for healthy controls.

| qMRI metrics | *N* | Baseline (SE) | Change [95%-CI] | *p*-value |
| --- | --- | --- | --- | --- |
| **Fat fraction (%)** | 209 | 4.8 (0.1) | +0.1 [0.1;0.2] | < 0.001* |
| Quadriceps |  | 4.4 (0.1) | +0.1 0.0;0.3] | 0.058 |
| Hamstrings |  | 5.2 (0.1) | +0.4 [0.2;0.5] | < 0.001* |
| Adductors |  | 6.2 (0.2) | +0.0 [-0.1;0.2] | 0.740 |
| Anterior Group |  | 4.3 (0.1) | +0.1 [-0.1;0.3] | 0.132 |
| Posterior Group |  | 4.2 (0.1) | +0.0 [-0.1;0.2] | 0.536 |
| **Water T2 (ms)** | 209 | 29.8 (0.1) | +0.1 [-0.1; 0.1] | 0.184 |
| Quadriceps |  | 29.9 (0.1) | +0.0 [-0.1;0.2] | 0.864 |
| Hamstrings |  | 29.3 (0.1) | +0.1 [-0.0;0.3] | < 0.001* |
| Adductors |  | 29.1 (0.1) | +0.2 [-0.0;0.4] | 0.050 |
| Anterior Group |  | 30.0 (0.1) | +0.1 [-0.2;0.1] | 0.265 |
| Posterior Group |  | 30.3 (0.1) | +0.0 [-0.2;0.1] | 0.831 |
| **FA** | 207 | 0.22 (0.00) | +0.00 [-0.01;0.00] | 0.006* |
| Quadriceps |  | 0.20 (0.00) | +0.00 [-0.01;0.00] | 0.376 |
| Hamstrings |  | 0.21 (0.00) | -0.01 [-0.01;0.00] | 0.124 |
| Adductors |  | 0.25 (0.00) | -0.00 [-0.01;0.01] | 0.725 |
| Anterior Group |  | 0.24 (0.00) | -0.00 [-0.01;0.00] | 0.156 |
| Posterior Group |  | 0.22 (0.00) | -0.01 [-0.01;-0.00] | 0.018* |
| **MD (10^-3^ mm^2^/s)** | 207 | 1.54 (0.01) | +0.01 [-0.00;0.01] | 0.097 |
| Quadriceps |  | 1.54 (0.01) | +0.01 [-0.01;0.03] | 0.259 |
| Hamstrings |  | 1.54 (0.01) | +0.01 [-0.00;0.03] | 0.130 |
| Adductors |  | 1.44 (0.01) | +0.01 [-0.01;0.03] | 0.246 |
| Anterior Group |  | 1.58 (0.01) | -0.00 [-0.02;0.02] | 0.831 |
| Posterior Group |  | 1.56 (0.01) | +0.00 [-0.02;0.01] | 0.973 |
| **AD (10^-3^ mm^2^/s)** | 207 | 1.92 (0.01) | -0.00 [-0.01;0.01] | 0.695 |
| Quadriceps |  | 1.88 (0.02) | -0.00 [-0.11;0.02] | 0.646 |
| Hamstrings |  | 1.89 (0.01) | +0.01 [-0.01;0.03] | 0.286 |
| Adductors |  | 1.87 (0.02) | +0.01 [-0.01;0.03] | 0.327 |
| Anterior Group |  | 2.00 (0.01) | -0.01 [-0.03;0.01] | 0.382 |
| Posterior Group |  | 1.94 (0.01) | -0.01 [-0.03;0.01] | 0.278 |
| **RD (10^-3^ mm^2^/s)** | 207 | 1.35 (0.01) | +0.01 [0.00; 0.01] | 0.043* |
| Quadriceps |  | 1.37 (0.01) | +0.01 [-0.01;0.02] | 0.329 |
| Hamstrings |  | 1.36 (0.01) | +0.01 [-0.00;0.03] | 0.132 |
| Adductors |  | 1.23 (0.01) | +0.01 [-0.01;-0.03] | 0.380 |
| Anterior Group |  | 1.36 (0.01) | +0.01 [-0.01;0.02] | 0.571 |
| Posterior Group |  | 1.37 (0.01) | +0.00 [-0.01;0.02] | 0.541 |
